# Isolation of Small SSEA-4-Positive Putative Stem Cells from the Ovarian Surface Epithelium of Adult Human Ovaries by Two Different Methods

**DOI:** 10.1155/2013/690415

**Published:** 2013-02-18

**Authors:** Irma Virant-Klun, Thomas Skutella, Matjaz Hren, Kristina Gruden, Branko Cvjeticanin, Andrej Vogler, Jasna Sinkovec

**Affiliations:** ^1^Department of Obstetrics and Gynecology, University Medical Centre Ljubljana, Slajmerjeva 3, 1000 Ljubljana, Slovenia; ^2^Institute for Anatomy and Cell Biology, University of Heidelberg, Im Neuenheimer Feld 307, 69120 Heidelberg, Germany; ^3^National Institute of Biology, Vecna Pot 111, 1000 Ljubljana, Slovenia; ^4^BioSistemika, Tehnoloski Park 24, 1000 Ljubljana, Slovenia

## Abstract

The adult ovarian surface epithelium has already been proposed as a source of stem cells and germinal cells in the literature, therefore it has been termed the “germinal epithelium”. At present more studies have confirmed the presence of stem cells expressing markers of pluripotency in adult mammalian ovaries, including humans. The aim of this study was to isolate a population of stem cells, based on the expression of pluripotency-related stage-specific embryonic antigen-4 (SSEA-4) from adult human ovarian surface epithelium by two different methods: magnetic-activated cell sorting and fluorescence-activated cell sorting. Both methods made it possible to isolate a similar, relatively homogenous population of small, SSEA-4-positive cells with diameters of up to 4 **μ**m from the suspension of cells retrieved by brushing of the ovarian cortex biopsies in reproductive-age and postmenopausal women and in women with premature ovarian failure. The immunocytochemistry and genetic analyses revealed that these small cells—putative stem cells—expressed some primordial germ cell and pluripotency-related markers and might be related to the *in vitro* development of oocyte-like cells expressing some oocyte-specific transcription factors in the presence of donated follicular fluid with substances important for oocyte growth and development. The stemness of these cells needs to be further researched.

## 1. Introduction

The idea that females of most mammalian species have lost the capacity for oocyte production at birth has been challenged by the finding that neonatal and adult mouse ovaries possess stem cells which can be successfully proliferated and confirmed *in vitro* [1–3]. Pacchiarotti et al. [2] found in neonatal and adult mouse ovaries two distinct populations of female germline stem cells with different diameters: cells with diameters of 10–15 *μ*m in the ovarian surface epithelium and cells with diameters of 50–60 *μ*m in the center of the follicular compartment. Zou et al. published research on the production of offspring from a female germline stem cell line derived from neonatal and adult mouse ovaries [3]. They established neonatal mouse female germline stem cells, showing normal karyotype and high telomerase activity, by immunomagnetic isolation and culture for more than 15 months. The female germline stem cells were also isolated from adult mice ovaries and cultured for more than 6 months. Similarly to Pacchiarrotti, Zou et al. also identified the female germline stem cells in ovarian surface epithelium of adult mouse ovaries. These female germline stem cells were infected with the GFP virus and transplanted into the ovaries of infertile mice. The transplanted cells underwent oogenesis and the mice produced offspring that carried the *GFP* transgene. These findings contribute to the basic research of ovarian stem cells, oogenesis, and a new understanding of the physiology of the mammalian ovary and showed that ovarian surface epithelium might be an important source of germinal stem cells in adult mouse ovaries.

 In addition to the mouse model, several related studies in humans also show that adult human ovarian surface epithelium might be a source of stem cells. Bukovsky et al. confirmed that oocyte-like cells may develop *in vitro* in ovarian cell cultures set up by adult ovarian surface epithelium scrapings in postmenopausal women [4]. Virant-Klun et al. further identified putative stem cells and the development of oocyte-like cells in cell cultures established by the ovarian surface epithelium scrapings in women with no naturally present follicles or oocytes, postmenopausal women, and women with premature ovarian failure [5–7]. All this research was also confirmed by Parte et al. [8] in adult human ovarian surface epithelium as well as in some other mammalian species, such as sheep and marmoset monkey. Moreover, White at al. [9] have recently published their finding of the existence of rare mitotically active cells—germline stem cells—with a gene expression profile that is consistent with primitive germ cells and which can be purified from adult ovarian cortical tissue by protocol based on fluorescence-activated cell sorting (FACS). They obtained viable DDX4 (VASA-) positive cells when using the COOH antibody. These cells were expanded for months *in vitro *and spontaneously developed into haploid oocyte-like cells with diameters of up to 35–50 *μ*m. When transplanted into human ovarian cortical biopsies, follicles containing oocytes formed after xenotransplantation into immunedeficient female mice. They concluded that ovaries of reproductive-age women possess rare mitotically active germ cells that can be proliferated *in vitro* and could generate oocytes *in vitro* and *in vivo*, although they did not precisely identify their real origin and position in the ovarian cortex. 

The isolation of a homogenous population of putative stem cells from adult human ovarian surface epithelium had never been established. Therefore the aim of this study was to isolate a relatively homogenous population of putative stem cells from the ovarian surface epithelium for the first time, specifically in the context of reproductive-age women and women with no naturally present, mature follicles or oocytes in their ovaries, that is, postmenopausal women and women with premature ovarian failure, by two different methods: magnetic-activated cell sorting (MACS) and fluorescence-activated cell sorting (FACS), to confirm the presence of common putative stem cells in all these groups of women and to provide a new methodology for further research of putative ovarian stem cells. 

## 2. Materials and Methods

The OSE layer of the ovarian cortex biopsy was scraped in three groups of women: (1) one reproductive-age woman aged 35 years; (2) two postmenopausal women aged 58 and 64 years; (3) two women with premature ovarian failure (POF) aged 21 and 31 years (with no mature follicles or oocytes in their ovaries). All women were surgically treated at the unit of gynecological surgery at our department for different gynecological reasons (e.g., removal of ovaries to prevent breast cancer, diagnostic biopsy of the ovaries for endometrial cancer or borderline ovarian cancer in one ovary, diagnostic ovarian brushing, and cortex biopsy in women with POF). In each woman a part of the ovarian cortex biopsy was sent to the histopathological service to routinely evaluate the histology (malignant tissue, folliculogenesis, oogenesis, and presence of ovarian surface epithelium) of tissue sections after haematoxylin-eosin (HE) and cytokeratin (CK) staining, as described elsewhere [6]. Another part of the cortex biopsy was used for research, and ovarian surface epithelium was scraped to isolate the SSEA-4-positive putative stem cells. This study was approved by the Slovenian Medical Ethical Committee (Ministry of Health of the Republic of Slovenia, no. 110/10/05). All women included in this study signed a written consent to participate voluntarily and to donate their ovarian tissue for research purposes. 

### 2.1. Ovarian Surface Epithelium Brushing

POF epithelial cell clusters from each woman were retrieved laparoscopically by softly brushing the ovarian surface epithelium of the whole ovaries with a fine brush, which was washed in a volume of 10 mL of sterile physiological solution at room temperature. In the laboratory the physiological solution containing the ovarian surface epithelium brushing was centrifuged, and the pellet was resuspended in 1 mL of DMEM/F-12 culture medium with phenol red, supplemented with 3.7 g/L NaHCO_3_, 1% penicillin/streptomycin, 0.5% gentamycin, and pH adjusted to 7.4 with 1 M NaOH. The drops of culture medium with the brushings were monitored under an inverted microscope (Nikon ECLIPSE TE2000-S, Japan) equipped with a Nikon Digital Sight DS-Ri1 camera at 200/400x magnification (Hoffmann illumination). In each woman a 0.3 cm^3^ biopsy of the ovarian cortex was retrieved. Immediately after surgical treatment each biopsy was carefully rinsed using a warmed, sterile physiological solution to remove as many blood cells as possible. The biopsy was then put on a wet, sterile gauze and promptly transferred to the lab. Once in the lab it was put in a volume of 3 mL culture medium DMEM/F-12 with phenol red, as described above. The ovarian surface epithelium was scraped using a sterile surgical blade (Romed, The Netherlands) in a flow hood. The droplets of the cell suspension were observed under the inverted microscope at conventional (200–400x) and higher (6000x) magnification (immersion objective, dic-Nomarski illumination). The cell suspension retrieved by biopsy brushing was used to isolate putative stem cells by magnetic-activated cell sorting (MACS) or fluorescence-activated cell sorting (FACS), based its on SSEA-4 surface antigen expression. 

### 2.2. Magnetic-Activated Cell Sorting of SSEA-4-Positive Putative Stem Cells

Firstly, the isolation buffer was prepared and cooled to 4°C (refrigerator): DPBS without Ca and Mg ions (Gibco/Invitrogen, USA) and adding 0.1% human serum albumin (HSA) and 2 mM EDTA. The monoclonal anti-human/mouse stage-specific embryonic antigen-4 (SSEA-4) antibodies (R&D Systems, USA) were biotinylated according to the manufacturer's instructions. Then a volume of 1.5 mL of cell suspension retrieved by ovarian surface epithelium brushing was centrifuged in a microcentrifuge for 10 minutes at 350 g (2800 rpm). The pellet was resuspended in 500 *μ*L of cold isolation buffer with an added 25 *μ*L of DSB-X biotin labeled SSEA-4 antibodies and incubated for 10 minutes at 2–8°C. Then the cell suspension was washed in 2 mL of cold isolation buffer by centrifugation at 350 g for 10 minutes. The pellet was resuspended in 1 mL of cold isolation buffer. Then 75 *μ*L of resuspended FlowComp Dynabeads 110.61D (Invitrogen, USA) were added to the cell suspension and incubated for 15 minutes at 2–8°C by permanent mixing (not vortexing). After that, the microcentrifuge tube was put on a magnet 123-21D (Invitrogen, USA) for at least 1 minute. The supernatant was carefully removed and rejected. The microcentrifuge tube was removed from the magnet, and the beads were gently resuspended in 1 mL of cold isolation buffer using a pipette. Then the microcentrifuge tube was put on the magnet again for at least 1 minute. The supernatant was carefully removed and rejected. The microcentrifuge tube was removed from the magnet and 1 mL of FlowComp Release Buffer (Invitrogen, USA) was added to release the cells from the beads. The suspension was incubated for 10 minutes at room temperature by permanent mixing. Then the suspension was mixed using a pipette, and the microcentrifuge tube was put on the magnet for at least 1 minute. The supernatant with cells without beads was transferred to a new microcentrifuge tube, which was put on the magnet for at least 1 minute to remove the remaining beads. The supernatant with cells without beads was transferred to a new microcentrifuge tube. Finally, 2 mL of cold isolation buffer was added to the supernatant, and the mixture was centrifuged for 10 minutes at 350 g. After centrifugation, the pellet was resuspended in 250 *μ*L of culture medium DMEM/F-12 with phenol red. 

### 2.3. Fluorescence-Activated Cell Sorting of SSEA-4-Positive Putative Stem Cells

The SSEA-4-positive cells were isolated from a brushed ovarian surface epithelium cell suspension using fluorescence-activated cell sorting (FACSAria, BD Biosciences, San Jose, CA). Briefly, 10^6^ cells were resuspended in phosphate-buffered saline (PBS) with 1% penicillin/streptomycin (Sigma Aldrich, St. Louis, Montana, P0781) and stained with anti-SSEA-4 antibodies (BD Pharmingen, clone MC813-70) conjugated with phycoerythrin (PE). The sample stained with an appropriate isotype control (PE Mouse IgG3, *κ*; BD Pharmingen) was examined in parallel. All antibodies were added at saturation concentrations, and the cells were incubated for 30 minutes in the dark then washed and resuspended for sorting in PBS with penicillin/streptomycin at a concentration of 2 × 10^6^ cells/mL. Sorted cells were collected in Dulbecco's Modified Eagle's Medium (DMEM)/Nutrient Mixture F-12 Ham with L-glutamine and 15 mM HEPES (Sigma Aldrich, D8900), supplemented with 3.7 g/L NaHCO_3_ (Sigma Aldrich, S5761), 1% penicillin/streptomycin, and 0.5% gentamycin (Sigma Aldrich, G1272), and then pH adjusted to 7.4 with 1 M NaOH. The sorted cells were cultured in a controlled CO_2_-incubator at 37°C and 6% CO_2_ in their surrounding air. 

### 2.4. Immunocytostaining on SSEA-4, OCT4A, and SOX-2 Expression

The cells to be stained were fixed in paraformaldehyde and permeabilized with Triton. Then they were incubated with fetal bovine serum to block the nonspecific binding sites. After that, the cells were incubated with fluorescein isothiocyanate-(FITC-) conjugated antibodies against SSEA-4 (BD Biosciences Pharmingen, San Diego, California, dilution 1 : 100), with primary mouse anti-OCT4 monoclonal antibodies (Millipore, Temecula, California, clone 2 7F9.2, diluted 1 : 100) or phycoerythrin-(PE-) conjugated antibodies against SOX-2 (BD Biosciences Pharmingen, San Diego, California, dilution 1 : 50) for 1 hour in the dark and at room temperature. After staining, the cells were washed with PBS, mounted using a mounting medium with DAPI (VECTASHIELD), and observed under a fluorescence microscope (ECLIPSE E600, Nikon, Tokyo, Japan). 

### 2.5. DAPI Staining of the Cell Nuclei

To monitor the cell nuclei, a small drop of the cell suspension was put into a drop of Vectashield mounting medium for fluorescence with DAPI (Vector Laboratories), incubated for 20 minutes at room temperature in the dark, and observed under a fluorescence microscope (Nikon ECLIPSE E-600 with a Nikon Digital Sight camera, magnifications of 200/400x).

### 2.6. Culture of Ovarian Brushings in the Presence of Follicular Fluid

The cortex biopsy brushings were cultured in supplemented DMEM/F-12 culture medium with added 20% (v/v) donated follicular fluid to provide an ovarian/follicular niche. For anticoagulation the follicular fluid, retrieved after the written consent of the donor, was used immediately after the removal of the oocytes (to be fertilized *in vitro*). It was centrifuged for 10 minutes at 2500 rpm. The supernatant was filtered through a sterile Sartorius Minisart 0.45 *μ*m filter to remove all the cells. The filtered supernatant was heat inactivated at 56°C for 45 minutes. Then it was aliquoted and stored at −20°C until use. Before use, an aliquot of follicular fluid was thawed at room temperature and added to the culture medium. To set up the ovarian surface epithelium cell culture, for each woman the cell suspension retrieved by ovarian cortex biopsy brushing was dropped into four 4-well sterile plastic dishes; 5 drops of cell suspension were put in each well with 250 *μ*L of preincubated culture medium with added follicular fluid. Cells were cultured for 1 month in a CO_2_-incubator at 37°C and 6% CO_2_ on a feeder layer of autologous ovarian fibroblasts. Each cell culture was monitored daily under an inverted microscope. 

### 2.7. Gene Expression Analyses of Small Putative Stem Cells by Microarrays and Biomark Real-Time Quantitative PCR (qPCR) System (Fluidigm)

#### 2.7.1. Gene Expression Analyses by Microarrays

Three samples of populations of SSEA-4-positive putative ovarian stem cells (OSC), isolated by FACS from three different ovarian cell cultures in three different patients, were analyzed by microarrays in comparison with the positive control-three samples of hESCs (ESC) of H1 cell line (WiCell, WI, USA) and the negative control-three samples of human adult (dermal) fibroblasts (FB). We were interested in expressions of the primordial germ cell-(PGC-) related genes *PRDM1 *(*BLIMP1*), *PRDM14*, *DPPA3 *(*STELLA*), *DAZL,* and pluripotency and ESC-related markers, including *OCT4A*, *SOX2*, *NANOG*, *TERT*, *SALL4*, *CDH1,* and *LEFTY1.*

 Samples were lysed using SuperAmp Lysis Buffer and stored at −20°C. When collected, the samples were analyzed by Miltenyi Biotec (Bergisch Gladbach, Germany) according to established protocols. First, the SuperAmp RNA amplification was performed. Amplified cDNA samples were quantified using the ND-1000 Spectrophotometer (NanoDrop Technologies, Wilmington, DE, USA). The integrity of cDNA was checked using the Agilent 2100 Bioanalyzer platform (Agilent Technologies, Wokingham, UK). The Cy3-labeled cDNAs were hybridized overnight to an Agilent Whole Human Genome Oligo Microarrays 8 × 60 K and washed. Fluorescence signals of the hybridized Agilent Microarrays were detected using Agilent's Microarray Scanner System (Agilent Technologies). The Agilent Feature Extraction Software was used to read out and process the microarray image files. Two differential gene expression analyses (DGAs) were performed: hESCs versus OSCs and fibroblasts versus OSCs to identify differently expressed transcripts. For determination of differential gene expression (DGA), derived output data files were further analyzed using the Rosette Resolver gene expression data analysis system (Rosetta Biosoftware). Data were preprocessed by normalization and correlation analysis. The normalized intensities were log2-transformed and served as the basis for further analysis. A Student's *t*-test was performed on each gene separately using normalized log2 intensity data. Statistical significance was set at *P* < 0.05. The genes selected as reliable candidates for the differentially expressed genes were required to show at least a 16-fold average expression difference (log ratio = 4) between the sample groups. 

#### 2.7.2. Gene Expression Analyses by Biomark Real-Time Quantitative PCR (qPCR)

To validate the microarray data, three samples of putative ovarian stem cells remaining after micrarray analysis (OSC1-3) were analyzed with a Biomark Real-Time quantitative PCR (qPCR) system (Fluidigm, San Francisco, CA, USA). In all samples the expressions of the most expressed PGC, pluripotency, and embryonic stem cell-related genes *DPPA3*, *OCT4A*, *LEFTY1*, *SALL4, *and *CDH1* (as confirmed by microarrays) and of the housekeeping gene *GAPDH*, which was used for normalization, were analyzed in comparisons with three samples of positive control—hESCs (ESC1-3), and three samples of negative control—human adult (dermal) fibroblasts (FB1-3). The inventoried TaqMan assays (20x, Applied Biosystems, Carlsbad, CA, USA) were pooled to a final concentration of 0.2x for each of the 8 assays. The cells were harvested directly into 9 *μ*L RT-PreAmp Master Mix (5.0 *μ*L CellsDirect 2x Reaction Mix (Invitrogen); 2.5 *μ*L 0.2x assay pool; 0.2 *μ*L RT/Taq Superscript III (Invitrogen); 1.3 *μ*L TE buffer). The harvested cells were immediately frozen and stored at −80°C. Cell lysis and sequence-specific reverse transcription were performed at 50°C for 15 minutes. The reverse transcriptase was inactivated by heating to 95°C for 2 minutes. Subsequently, in the same tube, cDNA went through limited sequence-specific amplification by denaturing at 95°C for 15 seconds, and annealing and amplification at 60°C for 4 minutes for 14 cycles. These preamplified products were diluted 5-fold prior to analysis with Universal PCR Master Mix and the inventoried TaqMan gene expression assays (ABI) in 96.96 Dynamic Arrays on a BioMark System. Each sample was analyzed in two technical replicates. Ct values were obtained from the BioMark System and were transferred to the GenEx software (MultiD, Göteborg, Sweden). The compared groups of samples were analyzed on heatmap and hierarchical clusterings (Ward's Algorithm, Euclidean Distance Measure) and principal component analysis (PCA). 

### 2.8. Single-Oocyte-Like Cell Gene Expression Analyses by Real-Time RT-PCR

The total RNA was extracted from twelve oocyte-like cells developed in the OSE cell cultures of women with POF, three nonfertilized mature (metaphase II) oocytes from the *in vitro* fertilization program (positive control), and 5 samples of human chondrocytes (negative control), reverse transcribed and then amplified using TaqMan PreAmp Cells-to-CT Kit (Applied Biosystems). The total RNA from the chondrocytes (negative control) was extracted using an RNeasy Mini Kit (Qiagen), treated with DNAse I (Invitrogen), and reverse transcribed with a High Capacity cDNA Archive Kit (Applied Biosystems). All samples were analyzed with TaqMan gene expression assays (Applied Biosystems): c-KIT, VASA, DMC1, SCP3, ZP1, ZP2, ZP3, OCT4A, FIGLA, ACTB, and GAPDH, with the latter two serving as internal reference genes. Real-time PCR reactions were performed on an ABI PRISM 7900 HT Sequence Detection System (Applied Biosystems) in 384-well plates. For each gene, a limit of detection (LOD) was determined based on the signal from the negative control samples. Quantitative results were calculated using the ddCt method, using the positive control sample OOCYTE3 as a calibrator sample. More about this is described in the Supplemental Material available online at http://dx.doi.org/10.1155/2013/690415. 

## 3. Results and Discussion

The haematoxylin-eosin staining revealed that all ovarian samples included in this study were healthy and did not possess any malignant tissue. In both women with POF there were no mature follicles or oocytes in the ovarian cortex tissue; in one woman several primordial follicles were observed, while in another one no follicles or oocytes were present in the ovarian cortex. After the cytokeratin staining it was clear that all women expressed continuously or partially distributed ovarian surface epithelium layers at their ovarian surfaces. 

### 3.1. Small Putative Stem Cells in Ovarian Surface Epithelium Brushings

**Figure 1:**
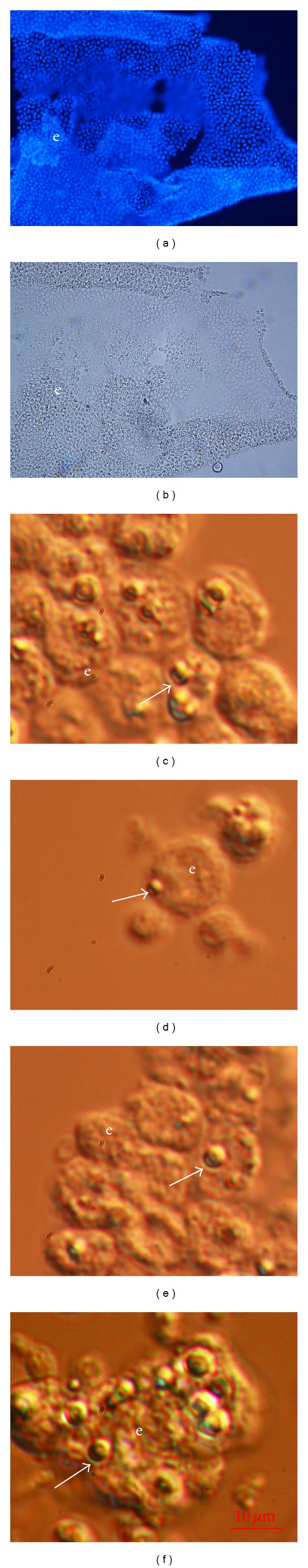
Ovarian surface epithelium brushings retrieved by laparoscopic brushing of the whole ovaries in women with premature ovarian failure. (a, b) Clusters of epithelial cells under a fluorescence and light microscope (blue: DAPI stained nuclei). (c–f) Small, round, yellow-coloured cells (arrows)—putative stem cells—attached to epithelial cells (inverted microscope, dic-Nomarski, 6000x magnification). e: epithelium. *Red Bar*: 10 *μ*m.

**Figure 2:**
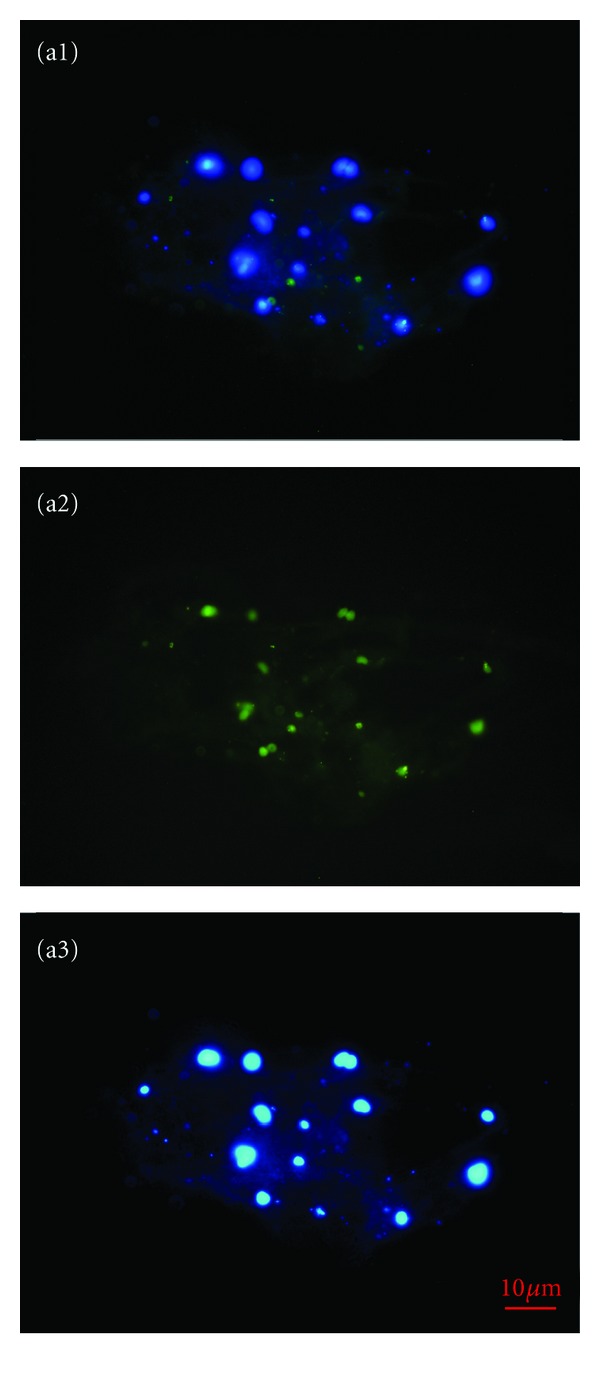
Small, round, SSEA-4-positive cells released from the ovarian surface epithelium brushings by enzymatic degradation. (a1) Merged. (a2) SSEA-4-positive (green) cells. (a3) DAPI (blue-) stained nuclei (fluorescence microscope, 400x magnification).

The laparoscopic brushing of the whole human ovaries (ovarian surface epithelium) was already performed in the past to study the production of steroids by human ovarian surface epithelial cells in culture and the possible role of progesterone as growth inhibitor [10]. In our study the ovarian surface epithelium was brushed to isolate putative stem cells from other cells and set up ovarian surface epithelium cell culture. In both patients with POF the surface epithelium brushings of the whole ovaries—large clusters of epithelial cells—were observed under an inverted microscope and under a fluorescence microscope after DAPI staining of the cell nuclei. Surprisingly, there were many small, round, yellow-coloured cells with diameters of 2–4 *μ*m captured among or attached to the epithelial cells (Figure 1). A low proportion of them had a larger diameter of up to 8 *μ*m. The possibility that smaller cells grew into bigger cells is not being dismissed. These cells appeared as small, yellow marbles in the ovarian surface epithelium brushings. They did not originate from blood and were an integral part of the ovarian surface epithelium; they also remained bound to the epithelial cells after several washings with a glass pipette. The small, round, yellow-coloured cells were visible under the inverted microscope and also under the conventional light microscope but were not seen after DAPI staining of the nuclei, presumably because they were too small. Similarly, this has been observed by Parte and colleagues [8] who experienced difficulty using DAPI staining of comparable small, round cells scraped from the human adult ovarian surface epithelium; they explained this by identifying a highly compacted nuclear chromatin which impeded staining. It was impossible to perform correct immunocytochemistry of these small cells bound to the epithelial cells. When attempting this, the small, round cells expressed a degree of autofluorescence. After their release from the epithelium by enzymatic digestion (collagenase and hyaluronidase), the nuclei of these cells were nicely stained by DAPI (Figure 2). This might be also related to the release from the ovarian (epithelial) niche, which plays an important role [11]. In the epithelial niche these cells might be in a dormant state, as has been published for some other types of stem cells [12]. These cells were also confirmed to be surface antigen stage-specific embryonic antigen-4- (SSEA-4-) positive (Figure 2). This is an established marker of pluripotency [13]; therefore the small, round, yellow-coloured cells among epithelial cells were proposed to be putative stem cells.

**Figure 3:**
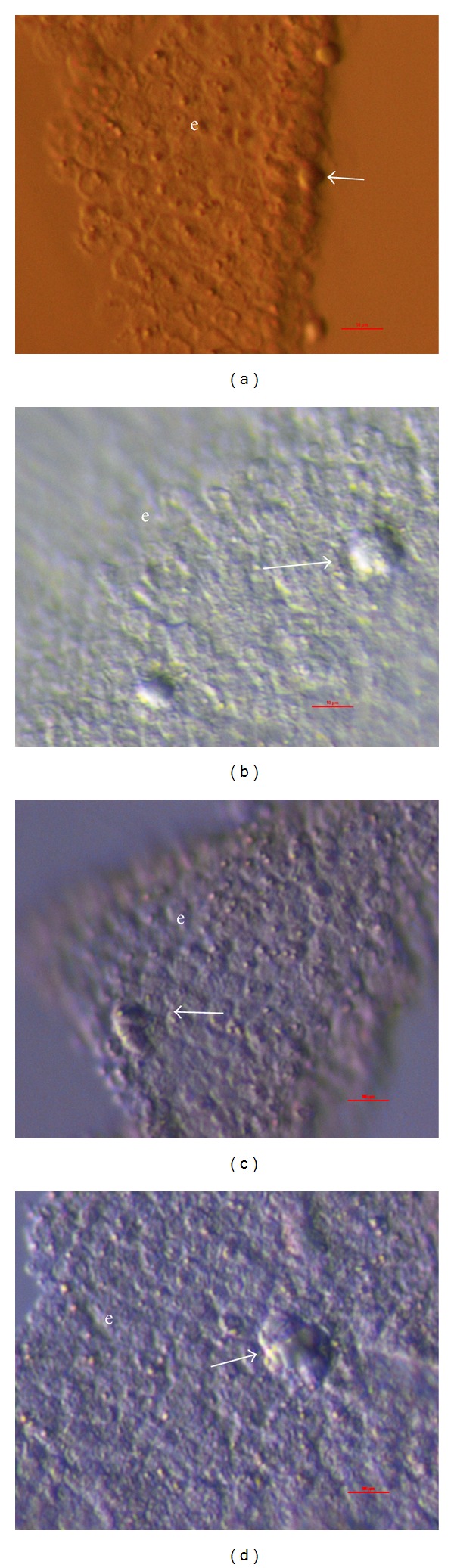
Ovarian surface epithelium brushed from the whole ovaries of women with premature ovarian failure and then cultured in the presence of donated follicular fluid; small, round cells are seen among epithelial cells. (a, b) Growing state of small, round cells (arrows) among epithelial cells. (c, d) Proliferating state of small, round cells (arrows) and formation of small clusters among epithelial cells (inverted microscope, Hoffman). e: epithelium. *Red Bar*: 10 *μ*m.

 A proportion of ovarian surface epithelium brushings were transferred into the DMEM/F-12 culture medium supplemented with follicular fluid. After approximately three days of culture the small, round, yellow-coloured cells began growing and reached a diameter of about 10 *μ*m (Figure 3). Some of these small cells proliferated, forming small clusters of cells that grew to a diameter of up to 15 *μ*m (Figure 3). When epithelial clusters were cultured in the same culture medium without added follicular fluid, the growth and proliferation of these small cells were not observed. The growth and proliferation of small, round cells among epithelial cells in the presence of follicular fluid might be induced by several important substances for oocyte growth and proliferation which are regularly present in the follicular fluid retrieved after controlled ovarian hyperstimulation.

### 3.2. Isolation of Putative Stem Cells by MACS and FACS

**Figure 4:**
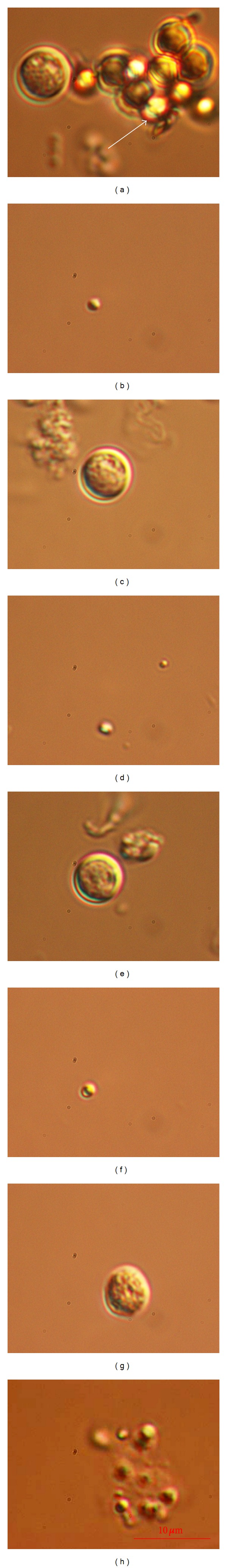
Two populations of SSEA-4-positive cells isolated by MACS from the suspensions of cells brushed from the ovarian cortex biopsies (ovarian surface epithelium). (a, c, e, and g) Bigger cells with diameters of up to 8 *μ*m and immunobeads (arrow). (b, d, f, and h) Small, round cells with diameters of up to 4 *μ*m (inverted microscope, dic-Nomarski, 6000x magnification). *Red Bar*: 10 *μ*m.

**Figure 5:**
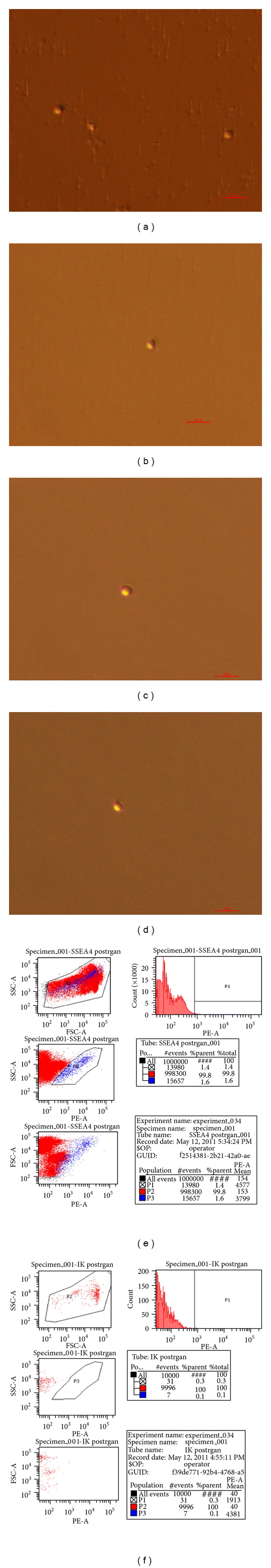
SSEA-4-positive cells isolated by FACS from the suspensions of cells brushed from the ovarian cortex biopsies (ovarian surface epithelium). (a–d) Small, round cells with diameters of up to 4 *μ*m (inverted microscope, Hoffman). *Red Bar*: 10 *μ*m. (e) FACS-sample; SSEA-4-positive cells represented 1.6% of all cells. (f) FACS-isotype control.

**Figure 6:**
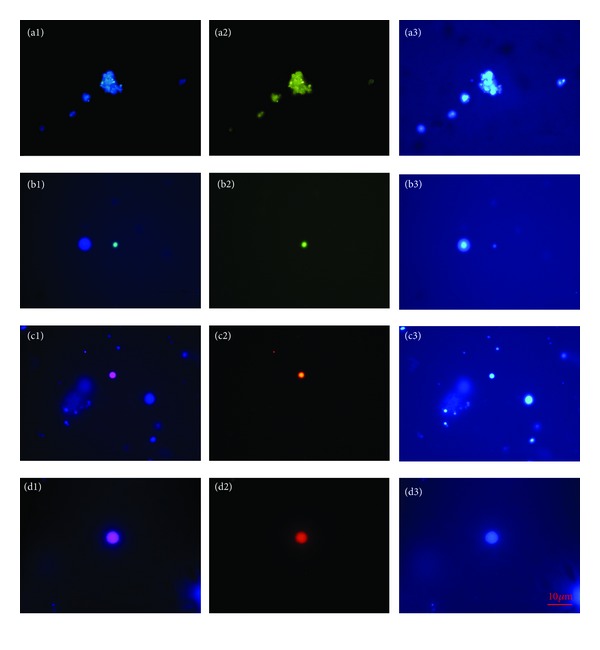
SSEA-4, OCT4A, and SOX-2-positivity of small, round cells isolated by MACS or FACS from the populations of cells retrieved by the ovarian cortex biopsy (ovarian surface epithelium) brushings, as revealed by immunocytochemistry. For SSEA-4, (a1) Merged; (a2) SSEA-4 (green) staining; (a3) DAPI (blue) staining of the cell nuclei. For OCT4A, (b1) Merged; (b2) OCT4A (green) staining; (b3) DAPI (blue) staining of the cell nuclei. For SOX-2, (c1, d1) Merged; (c2, d2) SOX-2 (red) staining; (c3, d3) DAPI (blue) staining of the cell nuclei (fluorescence microscope). *Red Bar*: 10 *μ*m.

After brushing the ovarian cortex biopsies, two different populations of SSEA-4-positive cells were isolated from cell suspensions by MACS: predominating small, round, yellow-coloured cells with diameters of 2–4 *μ*m and bigger, and yellow-coloured cells with diameters of up to 8 *μ*m (Figure 4); the bigger cells were not smooth, but expressed some dents on their surfaces. These small cells resembled those observed among epithelial cells in brushings retrieved from the whole ovaries (Figure 1) and were isolated from all ovarian samples. A similar population of small round, yellow-coloured cells with diameters of 2–4 *μ*m were also isolated by the FACS method from all ovarian samples (Figure 5). The SSEA-4-positive cells made up to 1.6% of the cells. The MACS and FACS-isolated cells were confirmed to be SSEA-4-positive by immunocytochemistry (Figure 6). Additionally, they expressed the pluripotency-related markers OCT4A and SOX-2 (Figure 6) [14]. Although a higher number of cells of similar dimensions and morphology were isolated by each method, only a small proportion of them expressed the analyzed markers of pluripotency, as can be seen in Figure 6. The isolated populations of cells were comparable to the ovarian surface epithelium putative stem cells previously observed by our group [5–7] and some other groups [8], but in this study they were isolated for the first time as a relatively homogenous population of SSEA-4-positive cells. Interestingly, White et al. [9] reported a comparable population of DDX4- (VASA-) positive cells using the FACS process; they found the cells with diameters of 5–8 *μ*m bound to the magnetic beads when they used the COOH antibody. In our study the isolation of a similar population of SSEA-4-positive cells by two different methods represents additional confirmation of the sorted cells and their possible stemness. The small putative stem cells from adult human surface epithelium have already been proposed to be very small embryonic-like stem cells (VSELs) [8], according to comparable small stem cells discovered by the Ratajczak group's research [15–17], and have been found in different adult human tissues and organs, such as bone marrow and umbilical cord blood [15–24]. It is too early to make a real conclusion about the character of small putative stem cells isolated from the adult human ovarian surface epithelium in this study. Our results showed that the small putative stem cells can be isolated not only from adult human ovarian surface epithelium of reproductive-age women but also from that of postmenopausal women and women with POF; therefore, they show an interesting prospect for the purpose of regenerative medicine in the future. 

### 3.3. Genetic Status of Small Putative Stem Cells

Microarray analysis showed that the primordial germ cell- (PGC-) related gene *PRDM1* (*BLIMP1*) was strongly expressed in small putative ovarian stem cells. In both hESCs (*P* = 8.29*E* − 03) and human adult fibroblasts (*P* = 2.71*E* − 04) *PRDM1* was significantly downregulated at the log ratio <−4 in comparison with ovarian stem cells, as revealed by DGAs. In addition putative ovarian stem cells expressed *PRDM14*, but the expression of this gene was upregulated in hESCs (*P* = 6.41*E* − 03) and down-regulated in fibroblasts (*P* = 6.94*E* − 03). The *PRDM1* gene is the key determinant of PGCs and plays a combinatorial role with *PRDM14* during PGC specification from postimplantation epiblast cells [25]. Moreover, *PRDM1* is a critical determinant of the germ cell lineage in mice. The genetic lineage-tracing experiments indicated that the PRDM1-positive cells originated from the proximal posterior epiblast cells and were indeed the lineage-restricted PGC precursors [26]. Therefore, the relation of small putative stem cells from the adult ovarian surface epithelium to PGCs is not excluded.

 Small putative ovarian stem cells weakly expressed the core genes related to pluripotency, ESCs, or germ cells, such as *DPPA3 *(*STELLA*), *OCT4A *(*POU5F1*), *SOX2,* and *NANOG*, which were significantly up-regulated in hESCs (*P* = 2.79*E* − 07, *P* = 9.90*E* − 03, *P* = 1.09*E* − 03, 1.24*E* − 02), yet the expression of genes *TERT* and *DAZL* was not detected in putative ovarian stem cells at all. The gene *TERT* may not be expressed in quiescent PGCs, and it has already been confirmed that it does not affect the proliferation and development of PGCs by itself [27]. Some recent studies also showed that the expression of the gene *DAZL* is not a prerequisite for PGC proliferation [28]. The expression of gene *DPPA3* may confirm the potential relation of small putative ovarian stem cells to PGC lineage [29].

**Figure 7:**
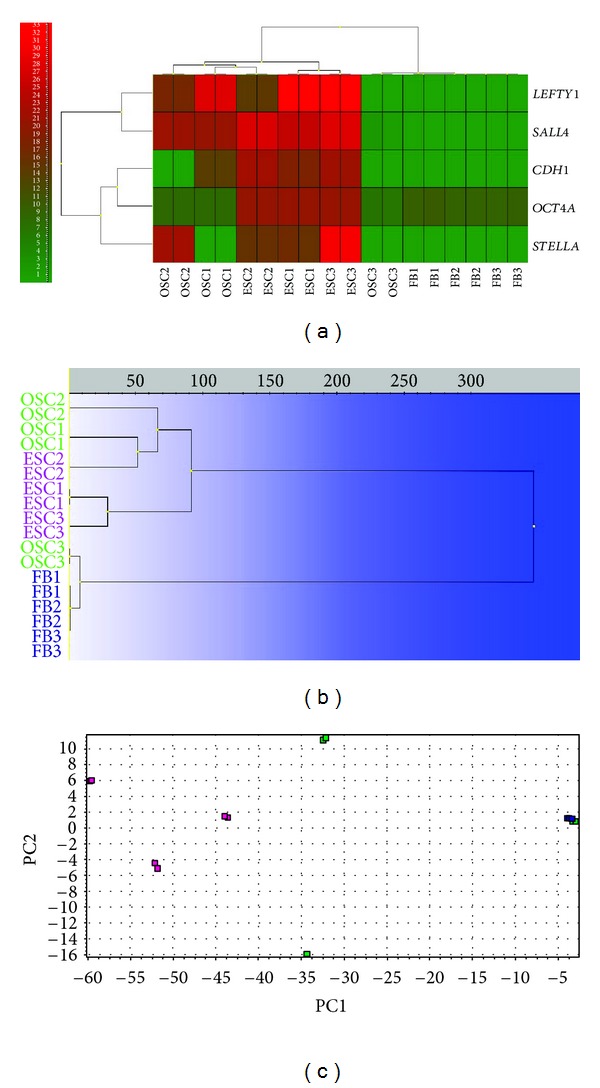
Expression of genes related to pluripotency and embryonic stem cells of putative ovarian stem cells (OSC1-3; green) in comparison with human embryonic stem cells (ESC1-3; violet) and human adult (dermal) fibroblasts (FB1-3; blue), revealed by RT-PCR (Fluidigm). Two OSC samples clustered with hESCs and one OSC sample clustered with fibroblasts. (a) Heatmap clustering. (b) Hierarchical clustering. (c) Principal component analysis (PCA).

Two of the samples of ovarian stem cells expressed the genes related to pluripotency more than the third one, as revealed by microarrays. When these two samples were compared to somatic fibroblasts, some of pluripotency-related genes were found to be significantly down-regulated in the somatic fibroblasts, such as *SALL4* (*P* = 2.23*E* − 03), *CDH1* (3.21*E* − 02), and *LEFTY1* (1.42*E* − 03); *P* values were adjusted to the effect of multiple testing according to Benjamini-Hochberg (limma, Bioconductor, programme R 2.14.1). Statistical significance was set at *P* < 0.05. These microarray data were also confirmed by Biomark Real-Time quantitative PCR (qPCR). Both the putative ovarian stem cells and hESCs strongly expressed all analyzed genes (*DPPA3*, *SALL4*, *CDH1, *and *LEFTY1*) related to pluripotency and ESCs, while human adult fibroblasts (FBs) only weakly expressed these genes or did not express them at all (Figure 7(a)). Two samples of putative ovarian stem cells (OSC1 and 2) clustered together with hESCs and were separated from fibroblasts (Figures 7(a)–7(c)), as revealed by heatmap, dendrogram, and principal component analysis (PCA). On the other hand, the other sample of putative ovarian stem cells (OSC3) clustered with fibroblasts, indicating the potential heterogeneity of these putative ovarian stem cell samples in terms of pluripotency. 

### 3.4. Development of Oocyte-Like Cells *In Vitro*

**Figure 8:**
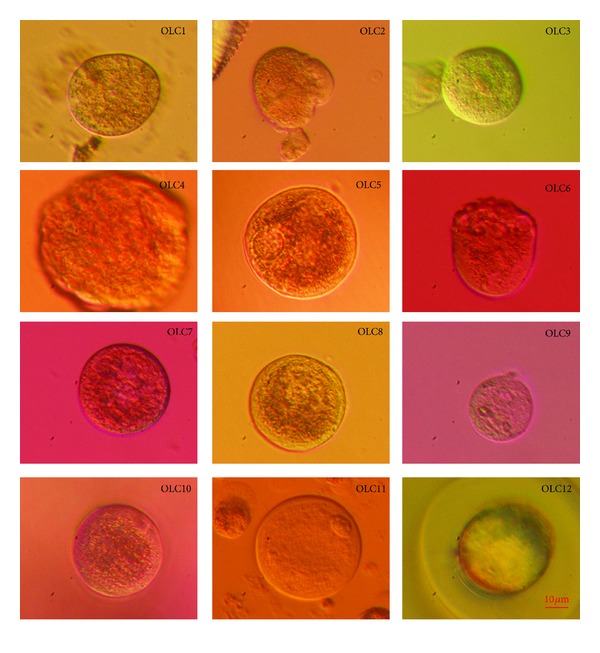
Twelve oocyte-like cells (OLCs) developed *in vitro* from putative ovarian stem cells (in two women with premature ovarian failure) cultured in a medium with added donated follicular fluid and analyzed by single-cell RT-PCR on the expression of genes related to pluripotency and oocytes. In OLC5 and OLC8 structures resembling germinal vesicles were observed; OLC8 expressed the meiosis-related gene *SCP3*. OLC12 developed a structure resembling zona pellucida (ZP) and indeed expressed the gene *ZP3* (inverted microscope, Hoffman). *Red Bar*: 10 *μ*m.

After MACS or FACS sorting, the isolated SSEA-4-positive cells were able to slowly proliferate and grow; however, they arrested once they reached diameters of up to 10–15 *μ*m. The limited *in vitro* proliferation and growth of these isolated cells may be related to the lack of an appropriate ovarian niche and a suboptimal culture system. In two women with POF the sorted SSEA-4-positive cells were transferred to autologous ovarian cell cultures, which had been previously set up by ovarian surface epithelium brushing in the same women. For each woman the sorted SSEA-4-positive cells were returned into two wells with culture medium supplemented with follicular fluid and with predominating autologous ovarian fibroblasts in a culture (free of bigger round cells). A proportion of small, round, yellow-coloured cells attached to the culture dish bottom (fibroblasts) and grew into bigger round cells with diameters of up to 60 *μ*m. Some of these cells had cytoplasm that could be compared to oocytes; therefore, we termed them oocyte-like cells. After 21–23 days of culture, 12 oocyte-like cells (Figure 8)—8 in one woman with POF and 4 in another one—were manually removed from the culture by a glass pipette and analyzed by single-cell RT-PCR on their expression of some genes related to pluripotency and oocytes. The majority of oocyte-like cells expressed OCT4A transcription factor (Table 1), which is known to be related to pluripotency [30]. In two oocyte-like cells the oocyte-specific transcription factor ZP3, related to zona pellucida, was expressed (Table 1). One of them (OLC12) expressed a zona pellucida-like structure, as can be seen in Figure 8. It has already been confirmed that ZP3 transcripts are expressed in the oocytes from early ovarian development, before the formation of follicles [31]. The function of ZP proteins during early fetal life is not clear, but the simultaneous expression of ZP3 suggests that it may have a role in the development of primordial follicles before zona pellucida formation. Moreover, in one oocyte-like cell the transcription factor SCP3 was expressed (Table 1); this transcription factor is related to the process of meiosis and is involved in the synaptonemal complex assembly and chromosome synapsis [32]. In this oocyte-like cell (OLC8) a structure resembling a germinal vesicle was observed, as can be seen in Figure 8. In one oocyte-like cell the c-KIT transcription factor was expressed (Table 1) that has already been proven to have an important role in follicle/oocyte development and germ cell proliferation [33]. On the other hand, nonfertilized mature oocytes from the *in vitro* fertilization programme expressed all of the analyzed genes related to pluripotency (*OCT4A*) and oocytes (*SCP3*, *ZP1*, *ZP2*, *ZP3*, *VASA*, *DMC1*, and *c-KIT*), while somatic chondrocytes did not express any of them. It may be concluded that the oocyte-like cells expressed a degree of pluripotency. In spite of that, they did not express some important oocyte-specific transcription factors, such as VASA and FIGLA, and were not real “oocytes” at this stage. The expression of oocyte-specific transcription factors ZP3, SCP3, and c-KIT might reflect the exposure of putative stem cells to donated follicular fluid added to the culture medium. Human follicular fluid contains several substances important for oocyte growth and maturation, such as estrogens, progesterone, FSH and androgens [34], proteins [35], amino acids [35], lipids (including free cholesterol and meiosis-activating sterol (FF-MAS)) [36, 37], and growth factors [38]. The oocyte-like cells developed in this study were not naturally present in the ovarian tissue because the female donors were characterized by severe POF. 

 The results of this study—the presence of putative stem cells in adult human ovaries—are in accordance with findings of White et al. [9], who in the journal *Nature* published the existence of rare mitotically active cells—germline stem cells—with a gene expression profile that is consistent with primitive germ cells in ovarian cortical tissue of reproductive-age women and with the findings of some other studies [1–8] which proposed human adult ovarian surface epithelium to be an important source of putative stem cells. Although, the previous study by White et al. showed that when human germinal stem cells were isolated from adult ovaries of reproductive-age women and cultured *in vitro* for up to 4 weeks, they formed actively dividing germ-cell colonies, expressed oocyte-specific markers, such as VASA, and resembled oogonia. In our study, even after 21 to 23 days of culture, oocyte-like cells expressed oocyte-specific genes such as *ZP3* or *SCP3*, but none of them expressed *VASA*. We suppose that the small SSEA-4-positive putative stem cells isolated from adult ovarian surface epithelium in this study more resembled PGCs, especially in terms of gene expression (e.g., overexpression of *PRDM1*), and might be the prestage of oogonia-like stem cells isolated by White et al. In our study small SSEA-4-putative ovarian stem cells expressed some of genes related to PGCs, pluripotency, and ESCs, and they were capable of growing into bigger oocyte-like cells, expressing some oocyte-specific genes in the presence of follicular fluid. But we do not exclude the possibility that these cells are capable of further development into fully competent oogonia or even oocytes *in vivo*, when in the natural and complex ovarian niche. 

## 4. Conclusion

Among epithelial cells of ovarian surface epithelium brushings, small, round cells with diameters of up to 4 *μ*m were observed. These cells were captured among epithelial cells or were attached to them. A similar population of small SSEA-4-positive cells was isolated from the adult human ovarian surface epithelium of functional and nonfunctional ovaries by two different methods: magnetic-activated cell sorting and fluorescence-activated cell sorting. These small, round, yellow-coloured cells expressed the analyzed markers of primordial germ cells (PRDM1, PRDM14, and DPPA3), pluripotency (OCT4A, SOX-2, SSEA-4, SALL4, CDH1, and LEFTY1) and might be related to *in vitro* development of oocyte-like cells, expressing some oocyte-specific markers (ZP3, SCP3, and c-KIT) in the presence of donated follicular fluid containing several substances important for oocyte growth and maturation. Based on these results, we propose that these small SSEA-4-positive cells are, in fact, putative stem cells and need to be further researched in terms of their potential use in reproductive and regenerative medicine in the future. 

## Supplementary Material

In the Supplementary Material the exact procedure of single-cell gene expression analyses of oocyte-like cells using real-time RT-PCR is described. Additionally, the exact results of gene expressions in oocyte-like cells developed in vitro from putative ovarian stem cells are provided in comparison with human oocytes (positive control) and human chondrocytes (negative control).

## Figures and Tables

**Table 1:** The expression of genes related to pluripotency (*OCT4A*) and oocytes (*c-KIT*, *DMC1*, *FIGLA*, *SCP3*, *VASA*, *ZP1*, *ZP2*, and *ZP3*) in twelve oocyte-like cells (OLC1–12) developed *in vitro* and analyzed by single-cell RT-PCR. The OLCs were compared to three nonfertilized mature (MII) oocytes (OOCYTE1–3) from the *in vitro* fertilization programme (positive control) and to 5 samples of human chondrocytes (H1–5; negative control). Eleven OLCs expressed the gene *OCT4A*, two OLCs the gene *ZP3,* one OLC the gene *SCP3*, and one OLC the gene *c-KIT*. The oocytes expressed all analyzed genes, while chondrocytes did not express any of them.

Sample name	Patient ID, age	* c-KIT*	*DMC1 *	*FIGLA *	*OCT4A *	*SCP3 *	*VASA *	*ZP1 *	*ZP2 *	*ZP3 *
OLC1	G.A., 22	−	−	−	**+**	−	−	−	−	−
OLC2	G.A., 23	−	−	−	**+**	−	−	−	−	−
OLC3	G.A., 23	−	−	−	**+**	−	−	−	−	−
OLC4	M.S., 21	−	−	−	**+**	−	−	−	−	−
OLC5	M.S., 21	−	−	−	**+**	−	−	−	−	**+**
OLC6	M.S., 21	−	−	−	−	−	−	−	−	−
OLC7	M.S., 21	−	−	−	**+**	−	−	−	−	−
OLC8	M.S., 21	−	−	−	**+**	**+**	−	−	−	−
OLC9	M.S., 21	**+**	−	−	**+**	−	−	−	−	−
OLC10	M.S., 21	−	−	−	**+**	−	−	−	−	−
OLC11	G.A., 28	−	−	−	**+**	−	−	−	−	−
OLC12	M.S., 25	−	−	−	**+**	−	−	−	−	**+**
OOCYTE1	IVF (PC)	**+**	**+**	**+**	**+**	**+**	**+**	**+**	**+**	**+**
OOCYTE2	IVF (PC)	**+**	**+**	**+**	**+**	**+**	**+**	**+**	**+**	**+**
OOCYTE3	IVF (PC)	**+**	**+**	**+**	**+**	**+**	**+**	**+**	**+**	**+**
H1	Cell culture (NC)	−	−	−	−	−	−	−	−	−
H2	Cell culture(NC)	−	−	−	−	−	−	−	−	−
H3	Cell culture (NC)	−	−	−	−	−	−	−	−	−
H4	Cell culture (NC)	−	−	−	−	−	−	−	−	−
H5	Cell culture (NC)	−	−	−	−	−	−	−	−	−
