# Supplementary material for: Isolation of Small SSEA-4-Positive Putative Stem Cells from the Ovarian Surface Epithelium of Adult Human Ovaries by Two Different Methods
Source: Biomed Res Int. 2013 Feb 18;2013:690415. doi: 10.1155/2013/690415 (PMC3590614; doi:10.1155/2013/690415)
Supplement: Supplementary file 1 — In the Supplementary Material the exact procedure of single-cell gene expression analyses of oocyte-like cells using real-time RT-PCR is described. Additionally, the exact results of gene expressions in oocyte-like cells developed in vitro from putative ovarian stem cells are provided in comparison with human oocytes (positive control) and human chondrocytes (negative control). [file 690415.f1.pdf]

Supplemental Experimental Procedure and Table

Single-cell gene expression analyses by real-time RT-PCR

Twelve oocyte-like cells and 3 non-fertilized MII oocytes from the *in vitro* fertilization programme (positive control) were mechanically removed from the culture in approximately 5 ml of culturing medium. One ml of of RNase inhibitor (Applied Biosystems) was immediately added to the sample and the sample was stored in liquid nitrogen until RNA extraction. Total RNA from stored cells was extracted, reverse transcribed and amplified using TaqMan® PreAmp Cells-to-CT™ Kit (Applied Biosystems) according to the manufacturer's protocol with the following modifications: DNAse I treatment of sample lysates was omitted, maximal recommended sample loads in all reactions were used, preamplification was performed with a pool of 12 TaqMan® gene expression assays (see below for list of genes) with ten cycles of amplification.

Five samples of human chondrocytes (approximately 30.000 cells per sample), used as negative control were washed and stored in liquid nitrogen. Total RNA was extracted from chondrocytes using RNeasy Mini Kit (Qiagen), according to the manufacturer's recommendations for animal cells. RNA was eluted with 35 ml of RNase free water heated to 65 °C with 10 min incubation at room temperature between the two elution steps and quantified spectrophotometrically (Nanodrop, Nanodrop Technologies). Samples were then treated with DNAse I (Invitrogen) and reverse transcribed with High Capacity cDNA Archive Kit (Applied Biosystems).

The following 11 TaqMan® gene expression assays were purchased from Applied Biosystems: c-KIT (Hs00174029_m1), VASA (Hs00251859_m1), DMC1 (Hs01095989_m1), SCP3 (Hs01041519_m1), (ZP1 Hs01399328_m1), ZP2

(Hs00185741_m1), ZP3 (Hs00610623_m1), OCT4A (Hs03005111_g1), FIGLA (Hs01079386_m1), ACTB (Hs99999903_m1) and GAPDH (Hs99999905_m1), the latter two serving as internal reference genes.

All assays (except OCT4A) include probes that span exon junctions and should therefore not amplify genomic DNA (gDNA). OCT4A TaqMan® gene expression assay was declared by its manufacturer (Applied Biosystems) as an amplicon that might amplify genomic DNA, probably because it might amplify other related pseudogenes that are known to exist. As a preventive measure all samples were tested for gDNA amplification by performing real-time PCR reactions on cell lysates that were diluted as they would be if the whole process of reverse transcription and preamplification would be carried out. No amplification was detected in any sample.

All real-time PCR reactions were performed on an ABI PRISM® 7900 HT Sequence Detection System (Applied Biosystems) in optical 384-well plates with optical adhesive covers (both Applied Biosystems) using universal cycling conditions (2 min at 50 °C, 10 min at 95 °C, followed by 45 cycles of 15 s at 95 °C and 1 min at 60 °C). Real-time PCR was performed in a final reaction volume of 10 μl containing 4.5 μl of pleamplified cDNA, or non-preamplified cDNA in the case of negative control samples, 1 x TaqMan® Gene Expression Assay and 1× TaqMan® Universal PCR Master Mix (both Applied Biosystems). Reactions were performed in two replicate wells in five-fold dilutions of samples. *ACTB* and *GAPDH* were used as internal reference genes.

The software SDS 2.3 (Applied Biosystems) was used for fluorescence acquisition and calculation of threshold cycles (Ct). For this calculation, the baseline

and the fluorescent threshold was set manually to intersect with the linear part of amplification curves of all amplicons, resulting in the final Ct value for each well.

Expression values were normalized to geometrical mean of *ACTB* and *GAPDH*. For each gene, a limit of detection (LOD) was determined based on the signal from negative control samples. Samples that had values above LOD were treated positive and samples below LOD (or without any amplification) were treated negative to that specific gene (marker). In addition, quantitative results were calculated using DDCt method (delta delta Ct), with a positive control sample OOCYTE3 as a calibrator sample.

Supplemental Table: Results of Single-Cell RT-PCR:

Sample Name	Patient ID, age	Expression of genes										
		ACTB	*GAPDH*	*C-KIT*	*DMC1*	*FIGLA*	*OCT 4A*	*SCP3*	*VASA*	*ZP1*	*ZP2*	*ZP3*
OLC1	G.A., 22	1.2	0.9	<LOD	<LOD	<LOD	**20.5**	<LOD	<LOD	<LOD	<LOD	<LOD
OLC2	G.A., 23	0.7	1.4	<LOD	<LOD	<LOD	**3.4**	<LOD	<LOD	<LOD	<LOD	<LOD
OLC3	G.A., 23	1.5	0.7	<LOD	<LOD	<LOD	**10.3**	<LOD	<LOD	<LOD	<LOD	<LOD
OLC4	M.S., 21	0.6	1.8	<LOD	<LOD	<LOD	**38.5**	<LOD	<LOD	<LOD	<LOD	<LOD
OLC5	M.S., 21	0.8	1.2	<LOD	<LOD	<LOD	**103.4**	<LOD	<LOD	<LOD	<LOD	**4677.1**
OLC6	M.S., 21	0.8	1.2	<LOD	<LOD	<LOD	<LOD	<LOD	<LOD	<LOD	<LOD	<LOD
OLC7	M.S., 21	0.7	1.5	<LOD	<LOD	<LOD	**165.3**	<LOD	<LOD	<LOD	<LOD	<LOD
OLC8	M.S., 21	0.6	1.7	<LOD	<LOD	<LOD	**67.8**	**752.6**	<LOD	<LOD	<LOD	<LOD
OLC9	M.S., 21	0.4	2.3	**189.1**	<LOD	<LOD	**17.3**	<LOD	<LOD	<LOD	<LOD	<LOD
OLC10	M.S., 21	0.6	1.6	<LOD	<LOD	<LOD	**12.9**	<LOD	<LOD	<LOD	<LOD	<LOD
OLC11	G.A., 28	<LOD*	1.6	<LOD	<LOD	<LOD	**0.0**	<LOD	<LOD	<LOD	<LOD	<LOD
OLC12	M.S., 25	0.8	1.2	<LOD	<LOD	<LOD	**6.4**	<LOD	<LOD	<LOD	<LOD	**547.9**
OOCYTE 1	IVF	0.8	1.3	**0.8**	**1.8**	**1.2**	**1.1**	**0.6**	**0.8**	**0.9**	**1.5**	**0.8**
OOCYTE 2	IVF	1.0	1.0	**0.9**	**0.8**	**0.5**	**2.0**	**0.5**	**0.9**	**0.5**	**1.3**	**0.9**
OOCYTE 3	IVF	1.0	1.0	**1.0**	**1.0**	**1.0**	**1.0**	**1.0**	**1.0**	**1.0**	**1.0**	**1.0**
H1	cell culture	0.9	1.1	<LOD	<LOD	<LOD	<LOD	<LOD	<LOD	<LOD	<LOD	<LOD
H2	cell culture	0.9	1.2	<LOD	<LOD	<LOD	<LOD	<LOD	<LOD	<LOD	<LOD	<LOD
H3	cell culture	0.8	1.2	<LOD	<LOD	<LOD	<LOD	<LOD	<LOD	<LOD	<LOD	<LOD
H4	cell culture	0.8	1.2	<LOD	<LOD	<LOD	<LOD	<LOD	<LOD	<LOD	<LOD	<LOD
H5	cell culture	0.8	1.3	<LOD	<LOD	<LOD	<LOD	<LOD	<LOD	<LOD	<LOD	<LOD
